# Suggested randomized, controlled trial for frovatriptan: a reply

**DOI:** 10.1007/s10194-011-0396-3

**Published:** 2011-10-21

**Authors:** Stefano Omboni, Lorenzo Pinessi, Lidia Savi, Brigida Fierro, Marco Bartolini, Carlo Lisotto, Giorgio Zanchin

**Affiliations:** 1 Italian Institute of Telemedicine, Varese, Italy; 2 Department of Neurology, University of Torino, Turin, Italy; 3 Department of Neurology and Psychiatry, University of Palermo, Palermo, Italy; 4 Department of Neuroscience, Polytechnic University of Marche, Ancona, Italy; 5 Ospedale Civile San Vito al Tagliamento, San Vito al Tagliamento, Italy; 6 Department of Neurology, University of Padova, Padua, Italy

Dear Sir,

We read with interest the comments of Dr. Tfelt-Hansen [1] regarding our three recently published randomized controlled trials comparing patients’ preference (primary endpoint) and efficacy (secondary endpoints) of frovatriptan 2.5 mg versus zolmitriptan 2.5 mg [2], rizatriptan 10 mg [3] and almotriptan 12.5 mg [4], and the meta-analysis of pooled individual data from the three studies [5]. In all studies frovatriptan showed similar preference and short-term efficacy outcomes (pain relief and pain-free episodes at 2 h) with respect to the other three triptans.

The questions put by Dr. Tfelt-Hansen sound appropriate. Doubts are raised on the usefulness of head-to-head preference trials of triptans and on the actual translation of their results into the clinical practice. We agree that patients may probably switch over time from one triptan to another because of individual preference, which might not be in line with results of randomized, controlled, comparative studies. However, the availability of results of head-to-head preference and efficacy trials may help physicians to make a first choice which might be very close to the actual patient’s preference. We should remind that some evidence on triptan preference in clinical practice does exist, even if we recognize that a tighter link between trials and clinical practice might be developed, for instance, by appropriate surveys [6, 7].

It is true that some guidelines usually refer to simple (and cheaper) oral analgesics and anti-emetics or pro-kinetic as the first line treatment of acute migraine, escalating to a (more expensive) triptan if this approach fails [8, 9]. This might sound reasonable in terms of the efficacy, because, as mentioned by Dr. Telft-Hansen, a meta-analysis showed that aspirin and sumatriptan act similarly in migraineurs [10]. More recently a publication from the same group showed a good efficacy of aspirin in treatment of acute migraine of moderate or severe intensity [11]. However, we think that a comparative study with sumatriptan, namely the oldest among triptans, might not be ideal, because newer triptans have been proved to be more effective than sumatriptan, with differences in the onset time of headache relief according to the characteristics of the studied triptan [12, 13]. Even though the efficacy of some triptans and aspirin might be similar in the acute phase of migraine, it is undisputable that triptans have a more definite place in treatment of chronic and recurrent migraine attacks in the most published guidelines. In addition, oral analgesics, like aspirin, are not an exempt from adverse events, as shown by a recent meta-analysis [14]. At the light of the current evidence we think that choice of use of non-steroidal anti-inflammatory-drugs or triptans for treatment of migraine headache should be based on several considerations, including characteristics of migraine, drug efficacy, patient’s preference and drug safety in the individual subject. Unfortunately, only a few of these aspects are taken into account in the current recommendations.
